# Cannabis use impacts pre‐stimulus neural activity in the visual cortices of people with HIV

**DOI:** 10.1002/hbm.25634

**Published:** 2021-08-31

**Authors:** Nicholas J. Christopher‐Hayes, Brandon J. Lew, Alex I. Wiesman, Mikki Schantell, Jennifer O'Neill, Pamela E. May, Susan Swindells, Tony W. Wilson

**Affiliations:** ^1^ Institute for Human Neuroscience Boys Town National Research Hospital Boys Town Nebraska USA; ^2^ College of Medicine University of Nebraska Medical Center (UNMC) Omaha Nebraska USA; ^3^ Montreal Neurological Institute McGill University Montréal Quebec Canada; ^4^ Department of Internal Medicine, Division of Infectious Diseases UNMC Omaha Nebraska USA; ^5^ Department of Neurological Sciences UNMC Omaha Nebraska USA

**Keywords:** gamma, magnetoencephalography, MEG, neural oscillations, neurocognitive decline, visuospatial processing

## Abstract

People with HIV (PWH) use cannabis at a higher rate than the general population, but the influence on neural activity is not well characterized. Cannabis use among PWH may have a beneficial effect, as neuroinflammation is known to be a critical problem in PWH and cannabis use has been associated with a reduction in proinflammatory markers. Thus, it is important to understand the net impact of cannabis use on brain and cognitive function in PWH. In this study, we collected magnetoencephalographic (MEG) brain imaging data on 81 participants split across four demographically matched groups (i.e., PWH using cannabis, controls using cannabis, non‐using PWH, and non‐using controls). Participants completed a visuospatial processing task during MEG. Time–frequency resolved voxel time series were extracted to identify the dynamics of oscillatory and pre‐stimulus baseline neural activity. Our results indicated strong theta (4–8 Hz), alpha (10–16 Hz), and gamma (62–72 Hz) visual oscillations in parietal–occipital brain regions across all participants. PWH exhibited significant behavioral deficits in visuospatial processing, as well as reduced theta oscillations and elevated pre‐stimulus gamma activity in visual cortices, all of which replicate prior work. Strikingly, chronic cannabis use was associated with a significant reduction in pre‐stimulus gamma activity in the visual cortices, such that PWH no longer statistically differed from controls. These results provide initial evidence that cannabis use may normalize some neural aberrations in PWH. This study fills an important gap in understanding the impact of cannabis use on brain and cognitive function in PWH.

## Graphical abstract

This work used a visuospatial processing paradigm in combination with magnetoencephalographic (MEG) imaging. Voxel‐level timeseries analyses were applied to study the impact of cannabis use on behavior and neural activity in people with HIV (PWH).

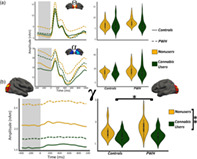

## 1 INTRODUCTION

For people with HIV (PWH), combined antiretroviral treatment (cART) can provide almost complete viral control, enabling many with optimal treatment adherence to experience healthy lives and normal life expectancy (Clifford & Ances, 2013; Samji et al., 2013). Yet, HIV‐related comorbidities remain a concern, with neurological comorbidities such as cognitive decline being among the most common (Clifford & Ances, 2013; Heaton et al., 2010; Roberts, Buckner, & Berman, 2010). These prevalent neurological comorbidities suggest continued neural changes related to inflammatory or other processes in PWH. Thus, additional treatment avenues targeting the CNS are of great interest. Cannabis is one such option that has been proposed in recent years, as it has been shown to have neuroprotective properties (Ellis et al., 2020; Manuzak et al., 2018; McCormick, 2018). Cannabis affects the CNS via cannabinoid receptors (Hall & Degenhardt, 2009; Skosnik, Cortes‐Briones, & Hajós, 2016) and can lead to large‐scale changes in neural population level dynamics (Skosnik & Cortes‐Briones, 2016). Importantly, it appears another major impact of cannabis on the CNS is anti‐inflammatory (Ellis et al., 2020; Manuzak et al., 2018; Molina et al., 2011). For example, Ellis et al. (2020) measured soluble cerebrospinal fluid biomarkers in PWH and analyzed the relationship of these markers with the recency of cannabis use. They found recent cannabis use was significantly associated with lower levels of proinflammatory markers, which supports the overall framework that cannabis may be used to control neuroinflammation in PWH. As per cognitive effects, the data in PWH are limited and inconclusive, especially regarding the impact of chronic use (vs. acute effects). A recent study found that chronic use was associated with cognitive decline on some assessments, but that there were no consistent patterns between lifetime cannabis use and cognitive dysfunction in PWH using cognitive screening assessments such as the Montreal Cognitive Assessment (MoCA); (Lorkiewicz et al., 2018).

Visuospatial processing is a central component of many higher order cognitive processes, and along with attention function is among the most common cognitive impairments observed in PWH (Antinori et al., 2007; Gorman, Foley, Ettenhofer, Hinkin, & van Gorp, 2009; Masters & Ances, 2014; Woods, Moore, Weber, & Grant, 2009). To interrogate the origin of these neuropsychological impairments, spatially precise brain imaging technologies such as magnetoencephalography (MEG) and structural and functional magnetic resonance imaging (sMRI/fMRI) have been used (Lew et al., 2021; Masters & Ances, 2014), and several studies have reported neural alterations in PWH, both inherent and related to visuospatial processing and attention function (Babiloni et al., 2012, 2014; Groff et al., 2020; Lew et al., 2018, 2020; Wiesman et al., 2018; Wilson et al., 2013). For example, fMRI studies of attention processing among PWH have shown decreases in parietal attention network regions compared with controls (Chang et al., 2004), and increases in prefrontal regions as a function of attention load (Chang, Yakupov, Nakama, Stokes, & Ernst, 2008). Similar fMRI studies focusing on the neural effects of cannabis use have found decreases in prefrontal and parietal regions in chronic cannabis users, accompanied by greater activation in frontal and occipital regions, with frontal changes normalizing as a function of abstinence (Chang, Yakupov, Cloak, & Ernst, 2006). Far fewer fMRI studies have investigated the combined effects of cannabis use and HIV. One study looked specifically at the effects on cognitive interference and found significant increases in left fronto‐insular cortex (Meade et al., 2019), whereas another inspected resting state connectivity and found significant disruptions in network organization (Hall, Lalee, Bell, Towe, & Meade, 2021).

MEG studies probing visual processing and attention dysfunction in PWH have reported aberrant theta power in middle and lateral prefrontal regions (Wilson et al., 2013), as well as occipital cortices (Wiesman et al., 2018). A recent study of selective attention processing in PWH and controls also found robust theta oscillatory activity across a network of brain regions, with theta interference activity in the left dorsolateral prefrontal cortex predicting performance on neuropsychological assessments of attention (Lew et al., 2018). Interestingly, prefrontal theta was selectively diminished in cognitively impaired PWH, but not controls or unimpaired PWH (Lew et al., 2018). These studies also found increased pre‐stimulus neural activity in the same brain regions serving visuospatial and attention processing, such that cognitively impaired and unimpaired PWH showed elevated theta, alpha, and gamma during the pre‐stimulus periods (Lew et al., 2018; Wiesman et al., 2018). Such altered neural oscillations and elevated pre‐stimulus neural activity have often been associated with concomitant impairments in task performance (e.g., Heinrichs‐Graham & Wilson, 2016; Lew et al., 2018, 2020; McDermott et al., 2019; Wiesman et al., 2018). Theta power has been strongly linked to initial stimulus registration and initialization and termination of attentional selection from one stimulus to another (Busch, Dubois, & VanRullen, 2009; Fries, 2015; Landau & Fries, 2012; Landau, Schreyer, van Pelt, & Fries, 2015), while alpha has been linked to the active inhibition of distracting visual input, often in the context of attention and/or working memory paradigms (Wiesman, Groff, & Wilson, 2019). In contrast, gamma activity has been most commonly associated with fine feature processing and integration, especially in visual cortex (Doesburg, Roggeveen, Kitajo, & Ward, 2008; Fries, 2015; Jensen, Gips, Bergmann, & Bonnefond, 2014; Muthukumaraswamy & Singh, 2013). Thus, by using MEG imaging, different components of visuospatial processing and attention can be at least partially distinguished temporally, spectrally, and spatially. The findings between cannabis and HIV research highlight an intriguing degree of overlap in the systems underlying visuospatial processing. In contrast to the increases in HIV, several studies have demonstrated gamma‐specific power decreases in visual processing systems of cannabis users during active periods (Skosnik et al., 2012; Skosnik, Krishnan, D'Souza, Hetrick, & O'Donnell, 2014). These results suggest a preferential effect of cannabis on high frequency neural activity.

In the current study, we used a visuospatial processing task and MEG to examine the impact of chronic cannabis use on behavioral performance and neural activity in PWH and demographically matched controls. Our primary hypotheses were that PWH would exhibit impaired performance on the task relative to controls, and that cannabis use would reduce the severity of neurophysiological aberrations in PWH. In other words, we expected that PWH who use cannabis would exhibit neuronal responses more similar to control participants who do not use cannabis. Such findings would suggest that cannabis use has at least some beneficial effects in PWH.

## 2 METHODS

### 2.1 Participants

All participants were recruited from the greater Omaha area as part of a larger NIH‐funded initiative to study the impact of aging with HIV on brain function. This investigation was reviewed and approved by the Institutional Review Board at the University of Nebraska Medical Center. All participants provided written informed consent. PWH (*n* = 40) and controls (*n* = 41) were derived from the larger sample and further striated into the following four groups based on the criteria described below: PWH who regularly use cannabis (*n* = 18), PWH who do not use cannabis (*n* = 22), uninfected controls who regularly use cannabis (*n* = 21), and uninfected controls who do not use cannabis (*n* = 20). Demographic information is provided in Table 1. The four groups were matched on age, gender, race, and alcohol use (Audit‐C), but differed in education, and education was examined as a covariate in all analyses.

**TABLE 1:** Demographic, neuropsychological, behavioral, and clinical summary

	Control‐NU (20)	Control‐CU (21)	PWH‐NU (22)	PWH‐CU (18)	*p* value
*Demographics*					
Age (years)^a^	43.05 (10.38)	37.33 (9.86)	38.68 (10.30)	43.33 (12.91)	n.s.
Education (years)^a^	15.85 (1.30)	14.87 (1.89)	14.95 (2.08)	13.77 (1.95)	<.05*
Sex (F/M)^a^	9/11	9/12	4/18	4/14	n.s.
*Neuropsychology*					
Fine motor^b^	−0.02 (1.01)	−0.84 (1.00)	−0.50 (1.04)	−0.96 (1.19)	n.s.
Learning and memory^b^	0.40 (0.95)	−0.59 (0.81)	−0.39 (0.82)	−0.08 (1.09)	n.s.
Language^b^	−0.15 (0.99)	−0.17 (0.72)	−0.22 (0.78)	−0.29 (0.85)	n.s.
Attention^b^	0.45 (0.81)	0.19 (0.94)	0.13 (0.59)	0.14 (0.63)	n.s.
Executive function^b^	0.1 (0.87)	−0.60 (0.90)	−0.37 (1.11)	−0.43 (0.93)	n.s.
*Visuospatial task*					
Reaction time (ms)^b^ ^,^ ^c^	517.33 (66.00)	544.69 (58.30)	568.36 (47.43)	564.63 (67.26)	<.01**
Accuracy^b^	97.79 (3.05)	97.43 (2.86)	96.07 (3.91)	96.31 (4.28)	n.s.
*Substance use*					
Audit‐C^b^	2.45 (1.95)	3.23 (1.81)	2.40 (1.70)	2.72 (2.05)	n.s.
Cannabis frequency^d^	–	33.85 (17.04)	–	27.72 (20.75)	n.s.
*HIV markers*					
CD4 nadir^d^	–	–	282.63 (173.69)	323.00 (181.47)	n.s.
Current CD4^d^	–	–	906.09 (500.35)	771.77 (391.86)	n.s.
Years diagnosed	–	–	9.77 (7.22)	9.28 (6.17)	n.s.

*Note*: Neuropsychology scores are functional domain composite *z*‐scores standardized. *p < .05, **p < .01, n.s. = *p* > .05.

Abbreviations: CU, cannabis users; NU, nonusers.

^a^ One‐way ANOVA/Chi‐square.

^b^ 2 × 2 ANOVA.

^c^ Main effect of HIV.

^d^ *t‐*test/Wilcoxon.

Inclusion criteria were as follows: (a) for all groups: adults between the ages of 18–65 years and Beck Depression Inventory II (Beck, Steer, & Brown, 1996) score of 21 or lower; (b) for cannabis user groups: current recreational cannabis consumption at a frequency of 4×/month or higher with no other illicit substance use; (c) for cannabis nonusers: no current illicit substance use; (d) for HIV groups: on cART for at least 1 year with undetectable viremia at time of study. To limit acute cannabis intoxication affects, all cannabis users were told to abstain from use on the day of their visit. Users who reported day‐of use were rescheduled. For all participants, exclusion criteria included neurological or psychiatric disorders (other than HIV‐related cognitive impairment), history of head trauma, hearing problems, or incomplete datasets. All participants had normal or corrected‐to‐normal vision.

### 2.2 Experimental visuospatial paradigm

**FIGURE 1:**
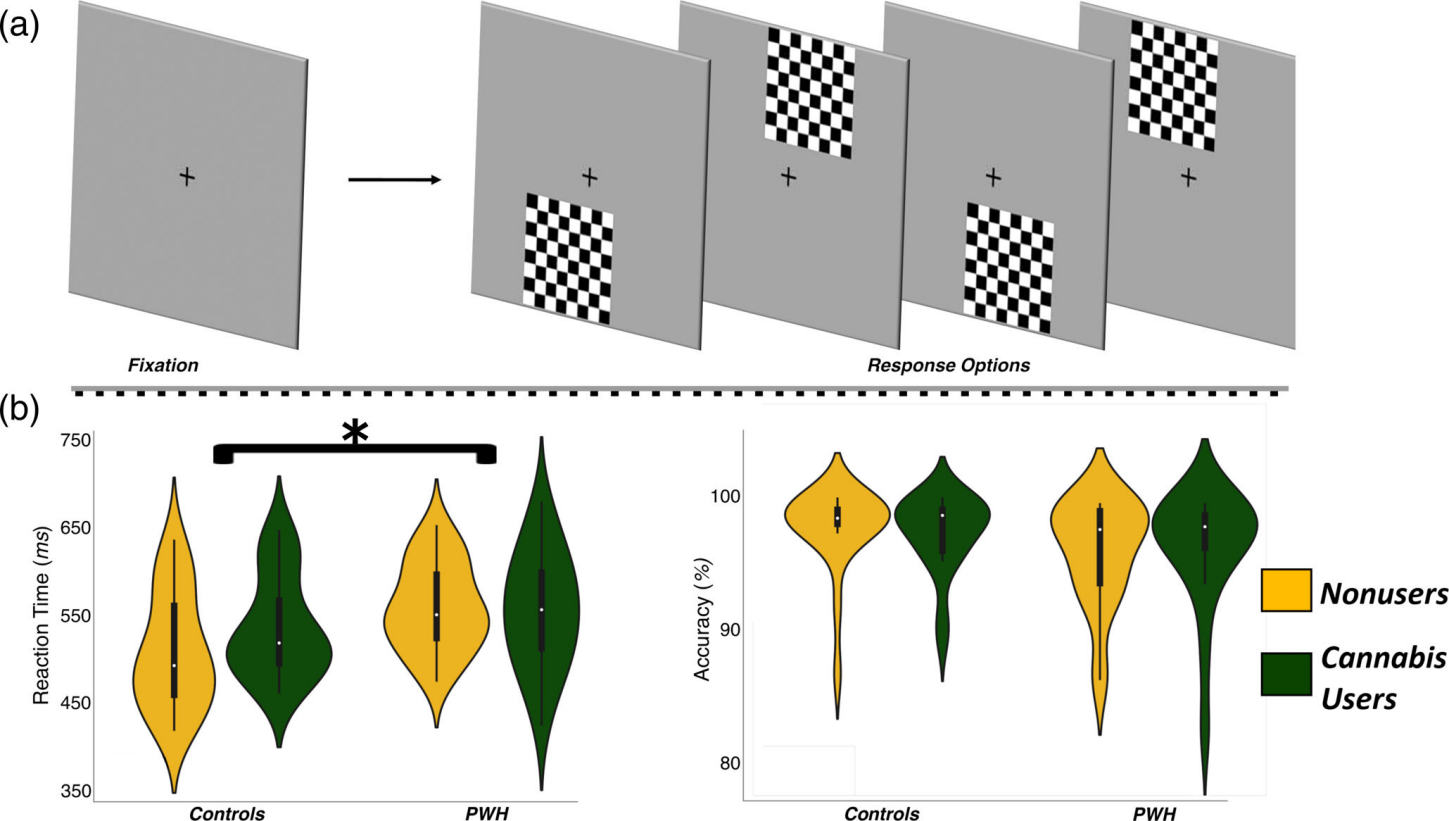
Experimental paradigm and behavioral results. (a) An illustration of the visuospatial task paradigm. Each trial was comprised of a fixation period lasting 2,000 ms (variable interstimulus interval: 1,900–2,100 ms) and a stimulus‐presentation period lasting 800 ms, which consisted of the appearance of a checkered grid in one of four locations. (b) Behavioral results from the visuospatial task; reaction time (in ms) is displayed on the *y*‐axis of the graph on the left, and accuracy (in % correct) is displayed on the *y*‐axis of the graph to the right. The color legend is shown to the far right (*p < .01)

The visuospatial discrimination task used in the present study was uniform to previously described reports (Wiesman, Heinrichs‐Graham, Proskovec, McDermott, & Wilson, 2017; Wiesman et al., 2018; Figure 1a). In short, the visual stimulus consisted of a black and white checkered grid, presented for 800 ms in one of four positions relative to a centrally presented crosshair: upper‐left, lower‐left, upper‐right, and lower‐right. Participants were instructed to respond to the location of the grid relative to the fixation with their index finger (upper‐left and lower‐left) or middle finger (upper‐right and lower‐right). Participants performed a total of 240 trials during the MEG recording. The stimuli subtended 42″ (horizontally) by 2″ (vertically) degrees of visual angle and were presented with the same equipment as in Wiesman, Christopher‐Hayes, Eastman, Heinrichs‐Graham, and Wilson (2021), Wiesman, Christopher‐Hayes, and Wilson (2021), and Wiesman and Wilson (2020). Reaction time and accuracy measures were collected and used as the primary behavioral measures.

### 2.3 Neuropsychological assessments

All participants underwent a battery of neuropsychological tests that assessed the following cognitive domains: Fine Motor (grooved pegboard: dominant/nondominant hands), Learning and Memory (Hopkins Verbal Learning Test—Revised: learning trials, delayed recall, and recognition), Language (phonemic and semantic verbal fluency), Attention (WAIS‐III: digit symbol coding and symbol search; Trail Making A), and Executive Function (Comalli Stroop: interference trial; Trail Making B). Raw scores per participant and assessment were converted to demographically adjusted *z*‐scores (Benedict, Schretlen, Groninger, & Brandt, 1998; Comalli, Wapner, & Werner, 1962; Heaton, Miller, Taylor, & Grant, 2004; Wechsler, 1997; Wilkinson & Roberson, 2006). Table 1 provides descriptive statistics for our neuropsychological metrics in each group.

### 2.4 MEG and MRI data acquisition

During data acquisition, participants were in constant contact with research personnel through real‐time audiovisual monitoring. Functional MEG recordings were collected using an Elekta/MEGIN MEG system (Helsinki, Finland) equipped with 306 sensors (204 planar gradiometers, 102 magnetometers). The system was in a one‐layer magnetically shielded room equipped with active shielding. The sampling rate was 1 kHz and an acquisition bandwidth of 0.1–330 Hz was used. Prior to MEG acquisition, four coils were attached to the subject's head and localized, together with fiducial and scalp surface points, using a three‐dimensional (3D) digitizer (FASTRAK 3SF0002, Polhemus Navigator Sciences, Colchester, Vermont). Once the subjects were positioned for MEG recording, an electric current with a unique frequency label (e.g., 322 Hz) was fed to each of the four coils. This induced a measurable magnetic field and allowed each coil to be localized in reference to the MEG sensors throughout the recording session. Structural T1‐weighted MRI were collected with a 3 T Philips Achieva MRI system with an eight‐channel head coil, consisting of one 3D 1 mm isotropic T1 FFE sequence (TR = 8.09 ms, TE = 3.7 ms, FOV = 240 × 240 mm, flip angle = 8°; see also Lew et al., 2020; Lew et al., 2021; Wiesman, Christopher‐Hayes, Eastman, et al., 2021; Wiesman, Christopher‐Hayes, & Wilson, 2021; Wiesman & Wilson, 2020).

### 2.5 MEG and MRI processing

MEG and MRI processing closely followed previously reported procedures (Wiesman, Christopher‐Hayes, Eastman, et al., 2021; Wiesman, Christopher‐Hayes, & Wilson, 2021; Wiesman & Wilson, 2020). MEG data were subjected to environmental noise reduction and corrected for head motion with MaxFilter software (Elekta/MEGIN, Helsinki, Finland) using the signal space separation method with a temporal extension (Taulu & Simola, 2006). Only data from the gradiometers were used for further analysis. All MEG and MRI data were further processed in BESA (Research: Version 7.0; MRI: Version 2.0; Statistics: Version 2.0). Structural MRI data underwent AC/PC alignment, inhomogeneity correction, segmentation, surface reconstruction, and transformation into standardized space. Each participant's MEG data were then co‐registered with their structural T1‐weighted MRI prior to analysis. Following source analysis (i.e., beamforming), each subject's functional MEG images were transformed into standardized space using the transform that was applied to the structural MRI volume and spatially resampled.

Additional noise variables (e.g., cardiac and blink artifacts) were regressed out of the MEG data using signal space projection (SSP; Uusitalo & Ilmoniemi, 1997). The resulting artifact‐corrected data were then bandpass filtered from 0.5 to 150 Hz, notch filtered at 60 Hz, and divided into 2,200 ms epochs. The baseline extended from −400 to 0 ms preceding the visual stimulus onset. Epochs containing other artifacts were excluded using an individualized fixed threshold method with a standard cutoff of 2.5 *SD*s from the individual's mean. Across all groups, the average amplitude threshold was 1,391.81 (*SD* = 562.22) fT/cm, the average gradient threshold was 980.58 (*SD* = 603.57) fT/(cm ms), and the average trials accepted per participant was 213 (*SD* = 12). None of the amplitude, gradient, or trial count values significantly differed by group, and therefore were not considered in later statistical comparisons.

### 2.6 MEG time–frequency transformation

Epochs were transformed into the time–frequency domain using complex demodulation (Papp & Ktonas, 1977; resolution: 2 Hz/25 ms; bandwidth: 4–100 Hz). The resulting spectral power estimations per sensor were averaged over trials to generate time–frequency plots of mean spectral density, which were normalized by the mean power during the −400 to 0 ms baseline time period. The time–frequency windows used for source imaging were determined by statistical analysis of the sensor‐level spectrograms across all participants.

Each data point in the spectrogram was initially evaluated using a mass univariate approach based on the general linear model. To reduce the risk of false positive results while maintaining reasonable sensitivity, a 2‐stage procedure was followed to control for Type‐1 error. In the first stage, paired‐sample *t*‐tests against baseline were conducted on each data point, and the output spectrogram of *t*‐values was thresholded at *p* < .05 to define time–frequency bins containing potentially significant oscillatory deviations across all participants. In stage two, time–frequency bins that survived the (*p* < .05) threshold were clustered. This clustering involved taking the temporally and/or spectrally neighboring bins that were also above the threshold (*p* < .05) and deriving a cluster value by summing the *t*‐values of all data points in the cluster.

Nonparametric cluster‐based permutation testing was then conducted using a Monte‐Carlo approach to randomly sample data points and re‐assign their active versus baseline data before recomputing the cluster sum values, which were eventually used to build a null distribution based on 1,000 permutations. The significance level of the observed clusters (from stage 1) was tested directly using this distribution (Ernst, 2004; Maris & Oostenveld, 2007). A detailed description of this approach is available (Wiesman et al., 2019; Wiesman, Christopher‐Hayes, & Wilson, 2021). Based on these analyses, the time–frequency windows that contained statistically significant oscillatory events across all participants and conditions were subjected to a beamforming analysis.

### 2.7 MEG source imaging

Source images reflecting neural activity for each statistically determined window were computed with a time–frequency resolved beamformer (Dalal, Sekihara, & Nagarajan, 2006; Gross et al., 2001; Van Veen, Van Drongelen, Yuchtman, & Suzuki, 1997). These source estimates were derived from the cross‐spectral densities of all combinations of MEG gradiometers averaged over each statistically determined time–frequency range of interest using active (i.e., post‐stimulus) and passive (i.e., pre‐stimulus) periods of equal duration and bandwidth (Hillebrand, Singh, Holliday, Furlong, & Barnes, 2005), and the solution of the forward problem for each location on a grid specified by input voxel space (resolution: 4 × 4 × 4 mm). Such images reflect baseline‐normalized source power for each voxel per participant. The resulting images were grand‐averaged across all participants for each time–frequency response and used to derive visual peak locations for virtual sensor extraction.

Virtual sensor data were computed by applying the sensor‐weighting matrix derived through the forward computation to the preprocessed signal vector, which yielded two orthogonal time series corresponding to the location of interest. Next, these virtual sensor data were decomposed into time–frequency space and vector summed to derive a single temporal envelope of the signal corresponding to the previously defined time–frequency window. This resulted in absolute amplitude and relative (baseline‐normalized) time series for each peak per participant. The absolute amplitude was used to examine differences in pre‐stimulus (i.e., baseline) neural activity, while the relative time series was used to assess differences in oscillatory response power.

Interactions and main effects were examined using a 2 (PWH vs. controls) × 2 (cannabis users vs. nonusers) between‐groups ANOVA model. Continuous demographic and alcohol use metrics were examined using a one‐way ANOVA. Pairwise comparisons were conducted using independent *t* and Wilcoxon tests. Categorical comparisons used Chi‐square tests (*χ* ^2^). Demographic, behavioral, neuropsychological, and time series statistical analyses were computed with SPSS and custom code with Matlab and *Statsmodels* (Python: Seabold & Perktold, 2010).

## 3 RESULTS

The four groups did not statistically differ on any demographic variable except education. Thus, all findings were analyzed with and without education as a covariate, however because it had no impact on our outcomes, we report statistical values without education included. There were no statistical differences between groups across neuropsychological domains. Finally, Audit‐C scores were similar across the four groups, and cannabis use frequency/amount did not differ between the two cannabis user groups (see Table 1).

### 3.1 Visuospatial task performance

For behavioral analyses, we examined reaction time and accuracy on the visuospatial discrimination task. A 2 × 2 ANOVA with HIV (infected/uninfected) and cannabis use (user/nonuser) as between‐subject factors indicated no significant effects on performance accuracy (*p*s > .36). As per reaction time, the same ANOVA design revealed that there was a significant main effect of HIV (*F*_1,77_ = 7.25, *p* = .008), but no main effect of cannabis use (*F*_1,77_ = 0.81, *p* = .37) and no HIV by cannabis interaction (*F*_1,77_ = 1.34, *p* = .25). The main effect of HIV reflected that PWH responded slower than controls. Post hoc analyses revealed that the cannabis using and non‐using PWH groups had similar reaction times on the task (*t*_38_ = 0.19, *p* = .85; Figure 1b).

### 3.2 Neural responses in parietal–occipital regions

**FIGURE 2:**
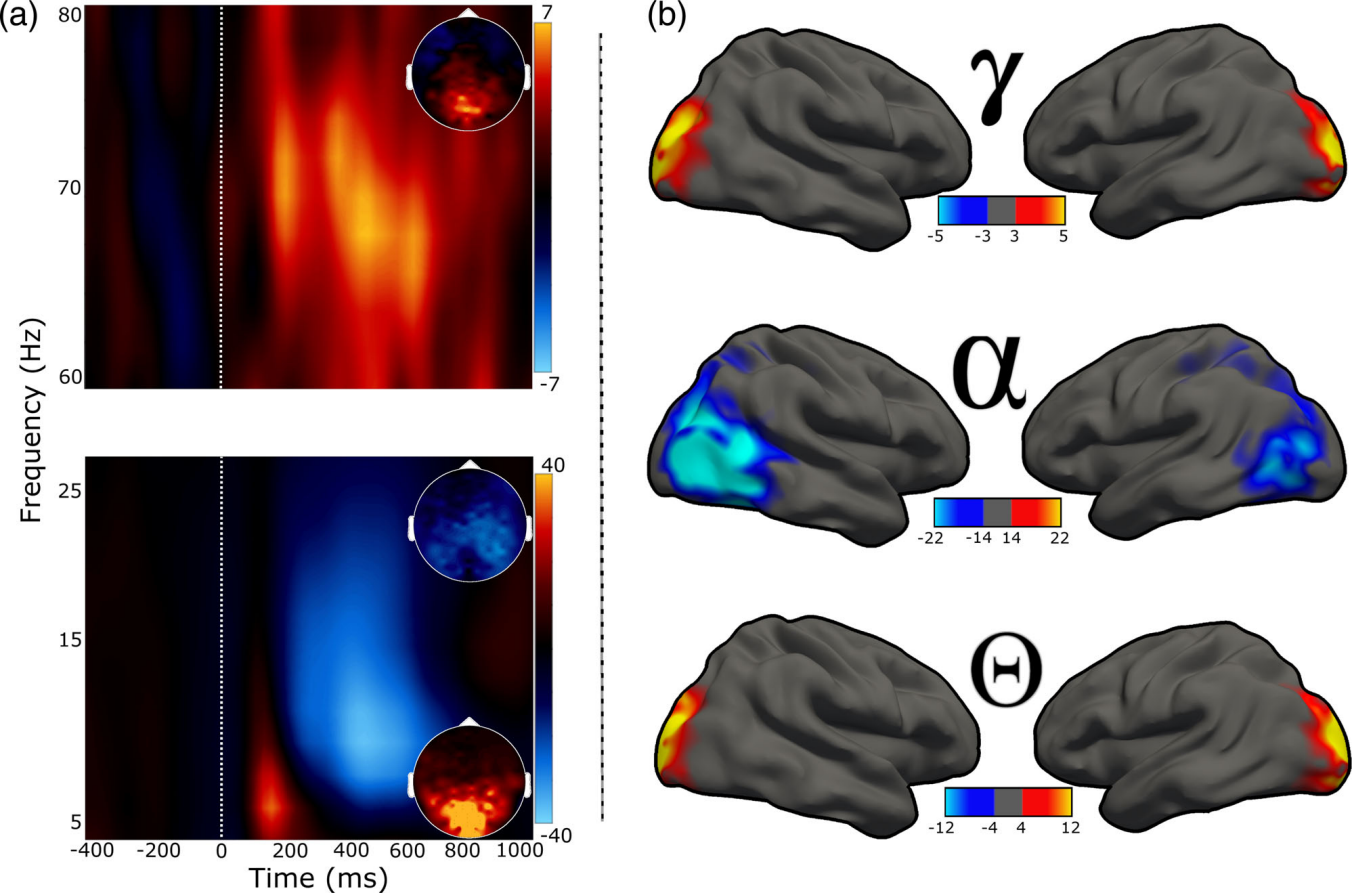
Neural responses serving visuospatial processing. (a): Spectrograms showing the three oscillatory responses identified at the MEG sensor level. Time (in ms) is denoted on the *x*‐axes, with 0 ms defined as the onset of the visual stimulus, and frequency (in Hz) is shown on the *y*‐axes. The lower spectrogram shows the theta (4–8 Hz, 25–275 ms) and alpha (8–14 Hz, 200–600 ms) responses, while the upper spectrogram shows the gamma (68–74 Hz, 200–600 ms) response. Insets show 2D power topographies of each oscillatory response grand‐averaged across the time–frequency windows of interest. (b): Activity from volumetric source images projected to the surface for visualization. Rows from top to bottom represent neural responses within the time and spectral windows of interest (i.e., gamma, alpha, theta). The color scale bars for spectrograms and 2D topographies (to the right of each plot), and for source maps (bottom of each plot) for each response reflects baseline‐normalized changes in power from the pre‐stimulus period

The time–frequency windows of interest were determined through a data‐driven statistical approach. In agreement with prior work using this visuospatial paradigm, responses were observed across three spectral windows (Figure 2a). Specifically, there was an early and transient increase in theta (4–8 Hz) between 25 and 275 ms, an extended increase in gamma activity (68–74 Hz) between 200 and 600 ms, and a power decrease in alpha (8–14 Hz) between 200 and 600 ms (*p*s < .001, corrected; Figure 2a). Note that the sensor‐level analyses focused on the period preceding the mean reaction time across all participants, as our goal was to focus on the neural oscillations underlying visuospatial processing.

To determine the cortical origin of the oscillatory responses identified at the MEG sensor level, the three time–frequency windows were imaged in each participant using a beamformer (see section 2.7). These images were then grand‐averaged across all participants, per oscillatory response, and the resulting images showed that increases in theta and gamma originated bilaterally in the posterior visual cortices, while the decrease in alpha power was centered on more lateralized parietal–occipital cortices (Figure 2b).

### 3.3 Group differences in multispectral oscillatory dynamics

For statistical analysis, we extracted the time series from the peak voxel of each cluster in the grand averaged maps (Figure 2b) per participant. Since all three oscillatory responses of interest were bilateral and we did not have any hypotheses related to laterality, the time series were averaged across hemisphere per oscillatory response per participant. This resulted in a single time series of response amplitude per oscillatory response (i.e., theta, alpha, and gamma) per participant. Next, to examine group differences in neural oscillations, we used the relative time series data (i.e., baseline‐normalized) and computed the average amplitude per participant within the time–frequency windows used for source imaging. To probe differences in pre‐stimulus neural activity (i.e., activity preceding stimulus onset), we used the absolute amplitude time series (i.e., not baseline corrected) and computed the average amplitude during the baseline period (i.e., −400 to 0 ms). Outliers were excluded at this stage based on a fixed threshold of 3 *SD* from the given timeseries window mean per group.

**FIGURE 3:**
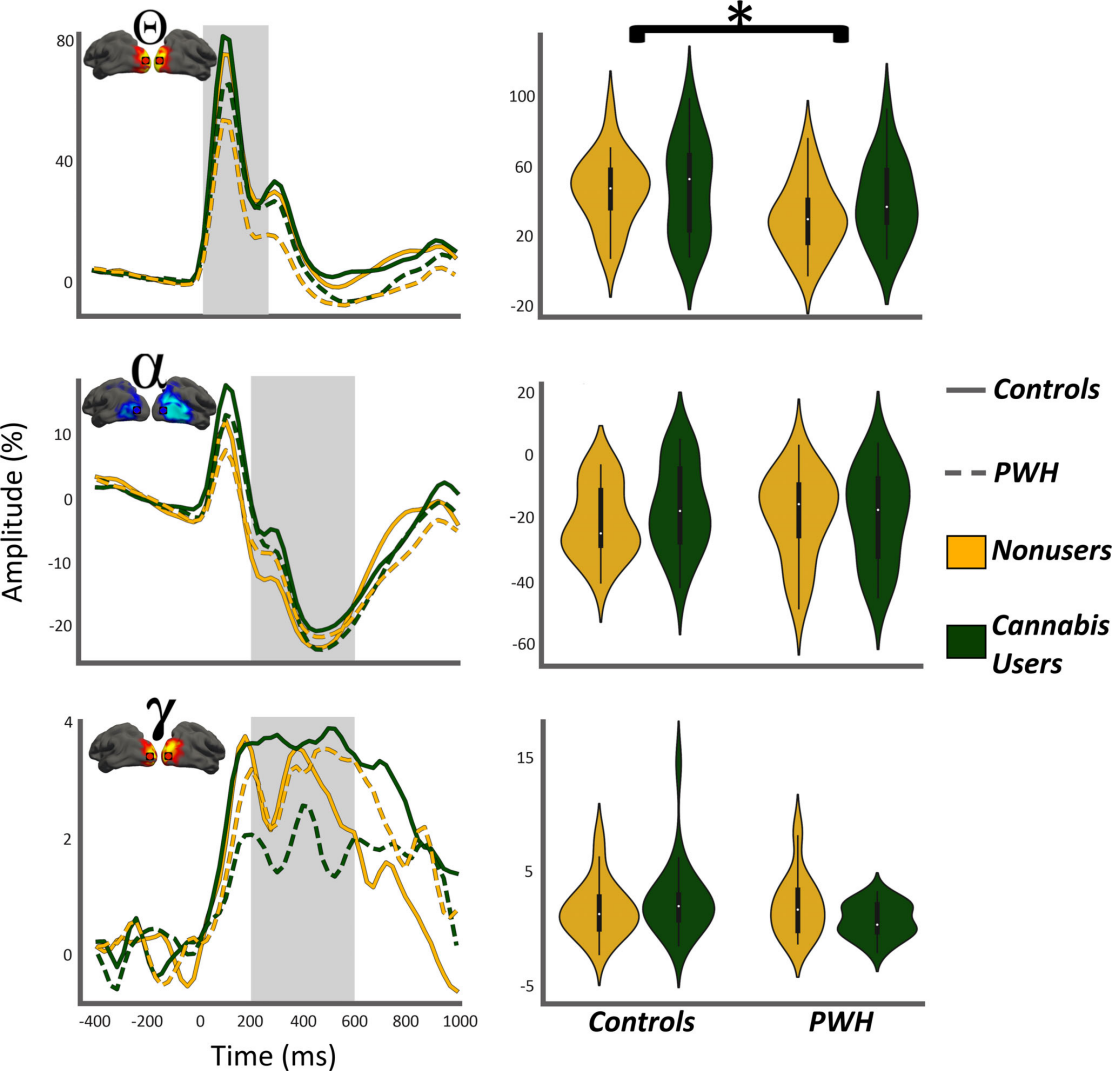
Oscillatory theta responses during visuospatial processing are weaker in PWH. (Left column): Time series from the peak voxel per oscillatory response collapsed across left and right hemispheric peaks in each participant and group averaged for visualization. The line features indicate group (legend on far right) and the gray highlighted area indicates the active window used to compute the means shown in the right column. In each plot, time (ms) is denoted on the *x*‐axis, with 0 ms defined as the onset of the visual stimulus, and the relative amplitude (in % from baseline) is denoted on the *y*‐axis. The inset shows functional images and location of peak voxel for improved clarity. (Right column): The means of each group computed from the gray highlighted area in the time series shown in the left column. Participant group is denoted via both the *x*‐axis and hue (*p < .05)

To evaluate the impact of HIV and cannabis on the neural oscillatory dynamics serving visuospatial processing, 2 × 2 ANOVAs were computed per response. We found no main effects or interactions for alpha (n.s.) or gamma (n.s.) responses. In contrast, we observed a significant main effect of HIV on theta oscillatory activity (*F*_1,75_ = 4.49, *p* = .03; Figure 3), but no main effect of cannabis or interaction. The main effect of HIV reflected reduced theta responses in PWH relative to controls.

**FIGURE 4:**
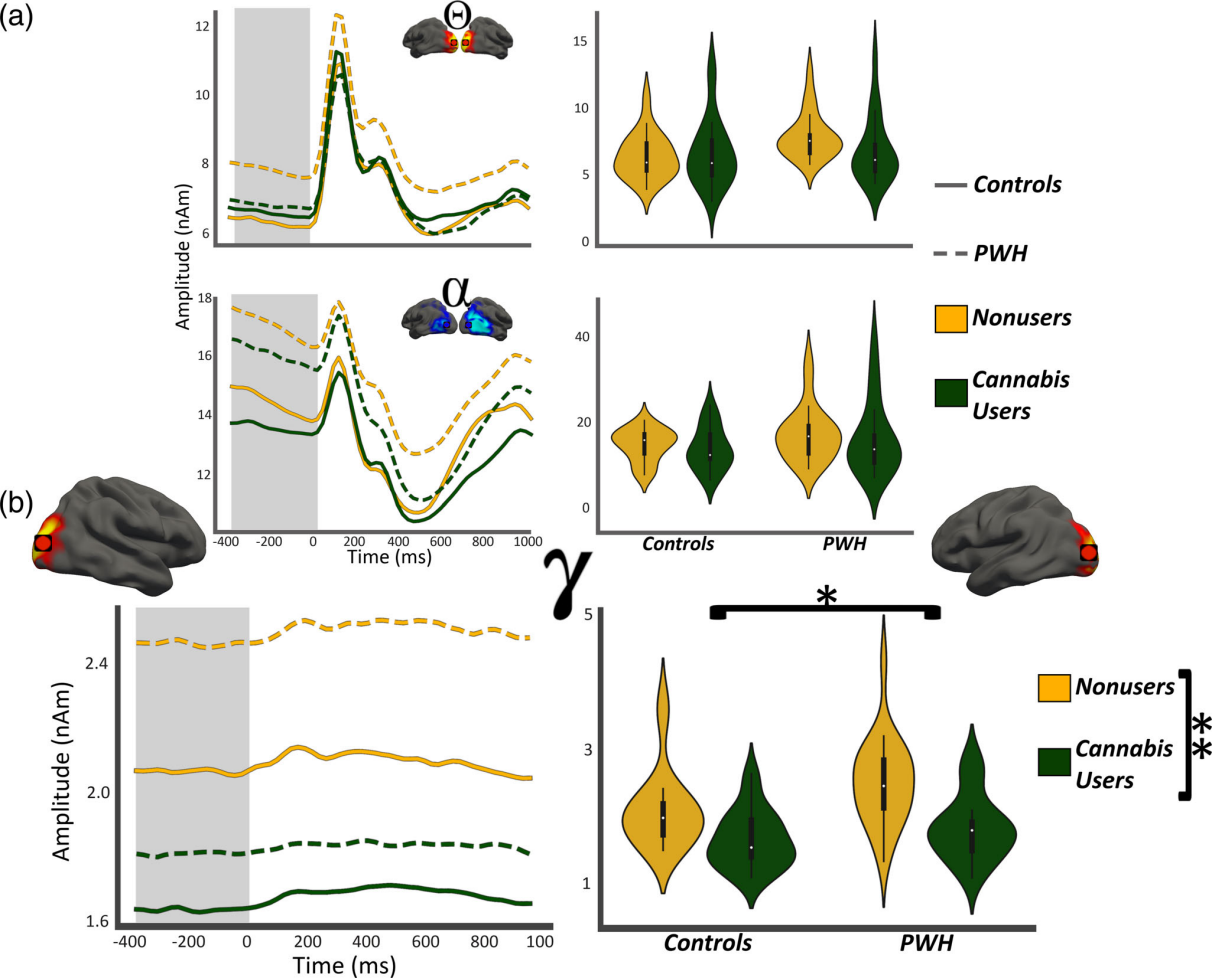
Gamma‐band neural activity was elevated in PWH and decreased in cannabis users. (Left column): Absolute amplitude time series from the same voxels shown in Figure 4 have been collapsed across hemisphere in each participant and group averaged for display. Line features indicate group and the gray highlighted area indicates the window of interest for computing neural activity during the baseline. Time (in ms) is denoted on the *x*‐axis, with 0 ms defined as the onset of the visual stimulus, and absolute amplitude (in nAm) is denoted on the *y*‐axis. The inset shows the location of the peak‐voxels. (Right column): The means of each group computed from the gray highlighted area in the left column per response. Participant group is denoted via both the *x*‐axis and hue. (a) Pre‐stimulus theta and alpha activity were not significantly affected by HIV or cannabis use, while (b) Pre‐stimulus gamma activity was affected by both. (*p < .05; **p < .001)

Regarding pre‐stimulus neural activity during the baseline period, we found no significant main or interaction effects in the theta (n.s.) or alpha (n.s.) range (Figure 4a). In contrast, significant main effects of both HIV (*F*_1,77_ = 6.19, *p* = .01) and cannabis (*F*_1,77_ = 21.37, *p* < .001; Figure 4b), but no interaction (*p* = .34) were observed for pre‐stimulus baseline gamma activity. The main effect of HIV reflected increased gamma activity in PWH relative to controls, whereas the main effect of cannabis reflected decreased pre‐stimulus gamma in cannabis users versus nonusers. Although the interaction effect was not significant, visual inspection of the pre‐stimulus gamma activity for each group (Figure 4b) suggested that cannabis use may have a unique effect on such activity in PWH. Thus, we performed a series of exploratory post hoc *t‐*tests. These tests revealed that non‐using PWH had significantly elevated pre‐stimulus gamma activity compared with cannabis using PWH (*t*_38_ = 3.71, *p* < .001) and both control groups (cannabis users: *t*_41_ = −5.16, *p* < .001; nonusers: *t*_40_ = −2.17, *p* = .03), while pre‐stimulus gamma activity did not significantly differ among cannabis using PWH and control users (*t*_37_ = −1.24, *p* = .22) nor control nonusers (*t*_36_ = 1.51, *p* = .14). Finally, cannabis using controls exhibited significantly reduced pre‐stimulus gamma activity compared with nonuser controls (*t*_39_ = 2.79, *p* = .008). Given the exploratory nature of these tests, we applied Bonferroni correction to control for multiple comparisons and all findings remained significant, except the difference between non‐using PWH and controls; thus, this difference should be interpreted with caution. In sum, cannabis use was associated with reduced gamma‐band activity in PWH and controls.

## 4 DISCUSSION

Herein, we used MEG neuroimaging and time series analyses to examine the impact of HIV and chronic cannabis use on the multispectral oscillatory dynamics underlying visuospatial processing and pre‐stimulus neural activity in the same brain regions. Importantly, this study was the first to examine the impact of chronic cannabis use on multispectral neural activity among PWH, which is an understudied area given the known role of neuroinflammation in PWH and the anti‐inflammatory properties of cannabis. Our results replicate previous findings showing that chronic cannabis use decreases gamma activity and had no significant impact on behavioral measures of visuospatial processing (Meade et al., 2019; Skosnik et al., 2012, 2014). Our findings also replicate work showing that PWH have aberrant theta oscillations in occipital cortices and behavioral impairments in visuospatial processing (Lew et al., 2018, 2020; Wiesman et al., 2018). Our results also significantly extend previous work by showing that cannabis use has a normalizing effect on the elevated pre‐stimulus gamma levels observed in PWH.

The most notable finding was the impact of cannabis use on pre‐stimulus gamma activity during the baseline period. Our post hoc analyses indicated that PWH who do not use cannabis have elevated pre‐stimulus gamma activity relative to controls, which replicates previous findings (Wiesman et al., 2018), while PWH who were chronic cannabis users exhibited pre‐stimulus gamma levels that were similar to controls. In short, cannabis use had a normalizing effect on pre‐stimulus gamma activity in PWH. These normalizing alterations were specific to the gamma frequency range, which is known to be necessary for the rapid and precise encoding of the fine‐grained features of visual stimuli (Fries, 2001; Fries, 2015; Müller, Gruber, & Keil, 2000; Muthukumaraswamy & Singh, 2013; Wiesman et al., 2017). Previous studies have associated elevated pre‐stimulus gamma with aging (Spooner, Wiesman, Proskovec, Heinrichs‐Graham, & Wilson, 2019), impaired processing in sensory (Spooner et al., 2018) and prefrontal cortices (Spooner et al., 2020), and CD4 nadir in PWH. Thus, the cannabis‐related reduction in pre‐stimulus gamma observed here in visual cortices likely improved neural processing in the PWH, but this will need to be tested directly in future studies. Of note, we conducted exploratory analyses examining a link between pre‐stimulus gamma activity and behavioral measures (reaction time or accuracy) and found no such relationship, but this could be due to the rather low‐level visual task implemented in this study. Future studies should employ more sensitive, higher‐level behavioral probes to ascertain the nature of any such relationship. Finally, cannabis use also decreased pre‐stimulus gamma activity in controls, such that the cannabis using control group had significantly weaker pre‐stimulus gamma compared with their non‐using peers. The functional impact of this decrease in terms of cognitive processing is not fully understood and future studies should probe the significance in terms of fine‐grained stimulus processing and visual attention. It may be the case that pre‐stimulus gamma below a specific level is also detrimental to visual processing, but this is speculation and will need to be directly tested.

In addition to our gamma findings, we replicated previous studies showing a significant reduction in visual theta responses and visuospatial processing speed in PWH (Wiesman et al., 2018). Interestingly, this reduction in theta oscillatory activity in PWH was not affected by cannabis use. Theta power has previously been tied to initial sampling of stimuli and the temporal organization of items in attention (Busch et al., 2009; Fries, 2015; Landau et al., 2015; Landau & Fries, 2012). Moreover, previous studies have linked theta activity in PWH to decreases in visuospatial processing accuracy, as well as attention and executive function when measured neuropsychologically (Lew et al., 2018; Wiesman et al., 2018). Thus, we replicate these findings and further show that they do not appear to be modulated by chronic cannabis use.

These findings provide further insight into the neural complexities and consequences of HIV, as well as the possible implications of comorbid cannabis abuse. Previous fMRI studies have investigated the combined effects of cannabis use and HIV (e.g., Meade et al., 2019; Hall et al., 2021), although these studies have not examined the spectral–temporal dynamics. Nonetheless, like our findings, these studies suggest that cannabis may have a positive impact on brain function in at least some regions in PWH, but that the overall impact is complicated and multifaceted. Previous MEG studies have investigated the spectral–temporal changes associated with HIV and with chronic cannabis use, albeit in separate studies that involved different experiments. Thus, by interrogating the neural–behavior relationships of cannabis in the context of PWH, this study further elucidates the systems of interest for treatment of the mechanistic and cognitive impacts of HIV.

Before closing, it is important to recognize the limitations of this study, and to identify future directions for this line of work. Although our study carefully controlled for demographic factors, alcohol consumption, and other important variables, cannabis use is inherently difficult to equate across a study group as there are different modes of use (smoking vs. ingested), a broad range of potency, and different frequencies of use even within a sample of chronic heavy users. Such variability is not ideal but reflects the current state of cannabis use research in the United States. Along these lines, future studies should employ a broad range of assessments to better characterize use patterns to the extent possible in their sample, as this is imperative to establishing links between cannabis use and specific neural markers (e.g., correlations with pre‐stimulus gamma). In addition, our visuospatial paradigm was minimally demanding and may not be suitable for the identification of higher order neural–behavior relationships (e.g., reaction time and gamma activity). Future studies examining cannabis and HIV should build on this design with other more challenging cognitive paradigms. Subsequent investigations should also examine whether the functional changes observed herein are accompanied by structural changes, although this will likely require larger samples as such differences are typically smaller. Despite these limitations, our results reinforce previously reported declines in visuospatial discrimination and altered neural dynamics among PWH, while also illuminating the impact of chronic cannabis use in PWH and a potential role for cannabis in modulating aberrant neural dynamics in people with HIV.

## Data Availability

The data that support the findings of this study are available from the principal investigators upon request.
